# Examining the impact of the COVID-19 pandemic on mental health among adults with type 2 diabetes: A systematic review of pre and during pandemic insights

**DOI:** 10.1097/MD.0000000000043112

**Published:** 2025-06-27

**Authors:** Amani Busili, Kanta Kumar, Laura Kudrna, Unaib Rabbani

**Affiliations:** a Department of Nursing and Midwifery, Nursing College, Jazan University, Jazan, Saudi Arabia; b Department of Nursing and Midwifery, College of Medical and Dental Sciences, University of Birmingham, Birmingham, United Kingdom; c Department of Nursing and Midwifery, Institute of Clinical Sciences, College of Medical and Dental Sciences, University of Birmingham, Birmingham, United Kingdom; d Applied Health Sciences, Institute of Applied Health Research, University of Birmingham, Birmingham, United Kingdom; e Family Medicine Academy, Qassim Health Cluster, Buraidah, Saudi Arabia.

**Keywords:** COVID-19 pandemic, mental health, systematic review, type 2 diabetes mellitus

## Abstract

**Background:**

The coronavirus disease 2019 (COVID-19) pandemic has posed significant challenges to mental health, especially among individuals with preexisting conditions such as type 2 diabetes. The interplay between chronic physical conditions and mental health is well-documented, yet the pandemic’s specific impact on the mental health of patients with type 2 diabetes remains underexplored. This study aimed to assess the prevalence of the most common mental health symptoms, including depression, anxiety, and insomnia, among type 2 diabetes patients before and during the COVID-19 pandemic to understand any potential changes in mental health outcomes.

**Methods:**

A systematic review of 42 observational studies published between 2010 and 2022 was conducted in accordance with PRISMA guidelines. Studies were identified through 5 electronic databases and included based on predefined eligibility criteria. Two reviewers independently screened and assessed the study quality. Due to limited directly comparable data, a narrative synthesis was performed.

**Results:**

The review included 42 studies, with 25 (60%) scoring 6 or less out of 9 in quality assessment. The prevalence of depression among type 2 diabetes patients ranged from 5.3% to 73.6% in prepandemic studies, compared with 5.6% to 30.4% during the pandemic. Anxiety prevalence was reported between 8.4% and 65.5% before the pandemic and remained at 8.4% during the pandemic. Insomnia was prevalent in 9.6% to 48.2% of patients’ prepandemic, with one study reporting a 31.4% prevalence during the pandemic. Two studies directly compared depression prevalence before and during the pandemic in the same population; one reported a significant increase from 19.3% to 30.4%, while the other found no difference.

**Conclusion:**

This study suggests that while the COVID-19 pandemic did not significantly exacerbate anxiety or insomnia in patients with type 2 diabetes, there may have been an increase in depression. The findings underscore the complexity of mental health outcomes during the pandemic and highlight the need for further research to fully understand the impact of COVID-19 on the mental health of individuals with type 2 diabetes. These results suggest the importance of ongoing mental health support for this vulnerable population.

## 1. Introduction

Globally, diabetes mellitus is one of the top 10 causes of death. Diabetes mellitus, along with cardiovascular diseases, cancers, and respiratory diseases, account for more than 80% of all premature noncommunicable disease deaths all over the world.^[1]^ Type 2 diabetes accounts for around 90% of all diabetes cases.^[2]^

While the physical complications of diabetes are well-documented, the impact of co-occurring psychiatric disorders is equally significant. When present alongside diabetes, these psychiatric conditions are associated with a variety of adverse outcomes, including reduced quality of life,^[3]^ increased healthcare costs,^[4]^ poor adherence to treatment,^[5]^ poor glucose control,^[6]^ an increase in emergency room visits due to diabetic ketoacidosis,^[7]^ a higher frequency of hospitalizations, and a higher level of absenteeism.^[8]^ In addition, there is a 2-fold higher level of care costs among patients with co-occurring psychiatric disorders and endocrine disorders than among those without co-occurring psychiatric disorders.^[9]^

In the COVID-19 pandemic, there were some studies indicating positive experiences associated with quarantine and lockdown measures implemented to curb the pandemic. These included the ability to spend more time with family, flexibility in working arrangements, and enjoying a less busy lifestyle.^[10]^ However, other studies suggested an increase in unhealthy behaviors,^[11]^ such as excessive television watching, heightened use of social media, and an uptick in the consumption of sweets and salty snacks, all of which can contribute to weight gain.^[12]^ Prolonged inactivity resulting from quarantine has the potential to impair the body’s ability to resist viral infections, thereby increasing the risk of immune, respiratory, cardiovascular, and musculoskeletal damage.^[13]^

Along with the physical consequences of a pandemic, there is a hidden, silent pandemic that accompanies the spread of infectious disease pandemics: the psychological effect.^[14]^ There was an elevated prevalence of depression, anxiety, and stress symptoms among various populations in the context of the pandemic, according to a recent review of the literature.^[15,16]^

Patients with chronic diseases, including those with type 2 diabetes, are more susceptible to the adverse effects and complications of COVID-19. Moreover, in patients with type 2 diabetes, COVID-19 on average increased severity risk by 2.3 times and mortality risk by 2.5 times.^[17]^ Furthermore, those patients face numerous challenges. These challenges appear as fear of emerging infectious diseases; grief caused by the infection of relatives and acquaintances; behavioral restrictions such as lockdown or quarantine to prevent disease spread; and anxiety related to the effect of their healthcare services during outbreaks.^[18]^

While the acute phase of the COVID-19 pandemic has concluded and precautionary measures have largely been lifted, it remains critical to investigate its long-term repercussions on vulnerable populations, particularly adults with type 2 diabetes. The mental health impact on these individuals is of significant concern, as the effects of the pandemic may persist long after the immediate threat has subsided. Despite numerous reviews that have explored the pandemic’s detrimental effects on mental health across various populations, there is a notable gap in the literature concerning a direct comparison of mental health outcomes in type 2 diabetic patients before and during the COVID-19 pandemic.

This systematic review seeks to address this gap by conducting a comprehensive analysis of the mental health impacts on type 2 diabetic patients, comparing studies from prepandemic and pandemic periods. By examining the prevalence of depression, anxiety, and insomnia, this review aims to provide critical insights into how the pandemic has affected this high-risk group. The findings from this review are essential for informing future healthcare strategies, ensuring the mental well-being of individuals with type 2 diabetes during and after global health crises, and preparing for future public health challenges.

## 2. Methods

This study is a systematic review of data from primary observational studies. The Preferred Reporting Items for Systematic Reviews and Meta-Analyses (PRISMA) guidelines were followed in conducting and reporting this review. The review protocol was registered with PROSPERO (Registration number CRD42022343639). Initially, the protocol included a meta-analysis to synthesize data from studies comparing outcomes before and during the COVID-19 pandemic. However, due to the limited number of studies directly comparing the same participants, the decision was made to exclude the meta-analysis and instead focus on a narrative synthesis. This change was implemented to maintain the methodological rigor and credibility of the review, considering the available data.

### 2.1. Search strategy

To achieve optimal searching in systematic review,^[19]^ 5 electronic bibliographic databases were used: Medline via PubMed, ISI Web of Knowledge via Web of Science, EMBASE, CINHAL, and PsycINFO between 2010 and June 2022.

The World Health Organization declared COVID-19 a pandemic on March 11, 2020. As a result, we classified studies with data collection after this date as being from the COVID-19 pandemic period. Conversely, data collection before this date was labeled as the prepandemic period. The only exception was when the authors explicitly stated their study took place during the pandemic. For instance, some studies^[20,21]^ were conducted before the official pandemic declaration, as the virus had started to spread earlier. Secondary searches in other sources such as Google Scholar were also conducted. The bibliographic software EndNote was used to store, organize, and manage all the references and ensure a systematic and comprehensive search.

First, we identified specific subject headings indexed in each database (such as MeSH terms, PsycINFO Thesaurus, and their synonyms). The Boolean operators “AND” and “OR” were used to combine search terms. In the following step, the search strategy combined MeSH terms and keywords that were used in MEDLINE (via PubMed). This approach was also adapted for other databases, ensuring that each search was tailored to the indexing and thesaurus terms specific to those platforms.

### 2.2. Inclusion criteria

All peer-reviewed observational studies examining the prevalence of common mental health problems—namely depression, anxiety, and insomnia—before and during the COVID-19 era among patients with type 2 diabetes were included. Studies were eligible to be included in the review if the study included type 2 diabetes adults (ages 18 years and older) with no gender, ethnicity, or setting restriction. Type 2 diabetes was diagnosed by self-report by physicians, medical records, or glucose testing (fasting plasma glucose 7.0 mmol/L and/or 2-hour postprandial plasma glucose 11.1 mmol/L).

### 2.3. Exclusion criteria

Studies were excluded if any of the following conditions were met: randomized controlled trials, nonrandomized controlled trials, qualitative studies, gray literature, editors, letters, comments, notes reviews, and meta-analysis; the study is not in English; studies measure the prevalence of mental health problems in war, human-made disasters, alcohol, or other drugs; studies carried out with children, adolescents, and pregnant women.

### 2.4. Types of outcomes and assessments

The outcomes included in this review included depression, anxiety, and insomnia. These outcomes were required to be assessed using validated tools such as the Patient Health Questionnaire, Beck Depression Inventory, Generalized Anxiety Disorder-7 (GAD-7), Insomnia Severity Index (ISI), or other validated scales.

### 2.5. Study selection

After the initial searches in the selected databases, the titles were checked and duplicate records were removed. This was followed by a screening of titles and abstracts to select articles for full-text review. Full-text articles were reviewed for eligibility and inclusion in the review. The disagreement at each stage was resolved by discussion between authors (A.B., U.R.).

### 2.6. Data extraction and synthesis

Data extraction was performed independently by 2 reviewers (A.B. and U.R.), who cross-checked each other’s data extraction. Any disagreements were resolved through discussion between them.

### 2.7. Risk of bias evaluation

The Joanna Briggs Institute (JBI) Critical Appraisal Checklists were used.^[22]^ This tool was specifically designed from observational studies reporting prevalence. The current systematic review was aimed at the prevalence of mental disorders, therefore, the JBI checklist was suitable. The JBI checklist items have 4 possible responses; no, yes, unclear, or not applicable, for the following items: representativeness of the sampling frame; appropriateness of the sampling method; appropriateness of sample size; adequateness of details of the sample and setting; sufficient analysis of data coverage; validity and reliability of the measures; appropriateness of reported statistics; and the adequateness of response rate. Articles were assigned one point per yes response. Articles with a score of less than 5 were considered to have a high risk of bias.^[23]^

## 3. Results

### 3.1. Search result

**Figure 1.**
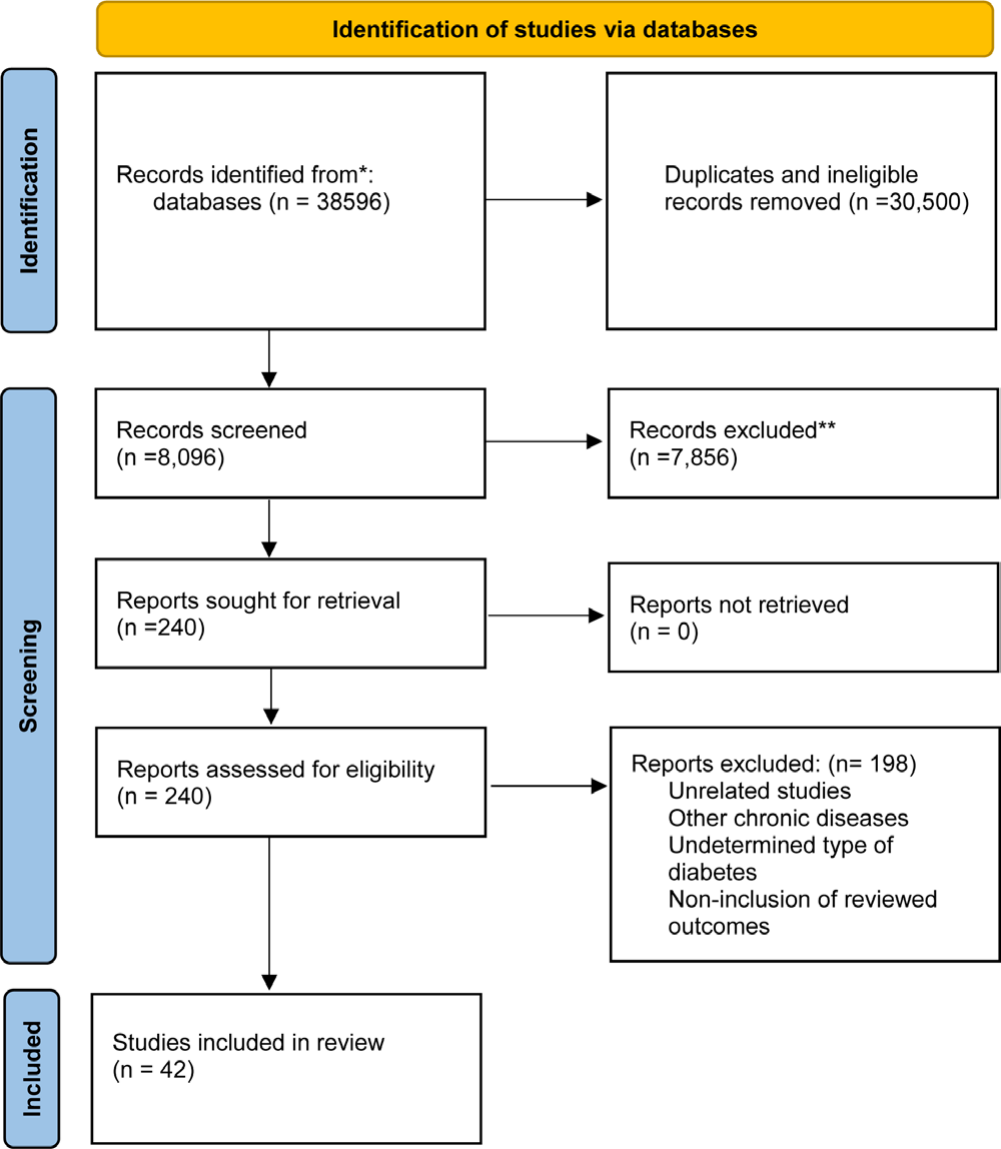
PRISMA diagram showing number of records at each step of review. PRISMA = Preferred Reporting Items for Systematic Reviews and Meta-Analyses. Adapted from Page et al,^[64]^ licensed under CC BY 4.0.

Following the PRISMA guidelines, this systematic review identified a total of 38,596 records from various databases. After initial screening and exclusion of duplicates and ineligible records, 8096 reports were sought for retrieval. Ultimately, 240 reports were assessed for eligibility, and 198 were excluded due to reasons such as unrelated studies, other chronic diseases, undetermined type of diabetes, and noninclusion of determined outcomes. The review included 42 studies that met the eligibility criteria^[20,21,24–63]^ (Fig. 1).

### 3.2. Risk of bias

We assessed the risk of bias in the included studies using JBI checklist. Out of a total possible score of 9, the quality scores ranged from 4 to 9. Only one (2.3%) study had a score less than 5 (high risk) while 57% (24/42) included studies had a score of 5 to 6 (moderate risk) out of a total score of 9 as detailed in Table 1.

**Table 1:** Characteristics and quality grade of included studies (N = 42).

Reference	Country	Title	Time*	Survey period	Study setting	Method of data collection	Sampling	Instruments and their cutoff values or diagnostic criteria	JBI Score
Khuwaja et al^[24]^	Pakistan	Anxiety and depression among outpatients with type 2 diabetes: a multicentre study of prevalence and associated factors	1	NA	Outpatient clinics	Self-administered	Nonrandom	Hospital Anxiety and Depression Scale (HADS)	7
Tovilla-Zárate et al^[25]^	Mexican	Prevalence of anxiety and depression among outpatients with type 2 diabetes in the Mexican population	1	January 2011–September 2011	Outpatients	Self-administered	Nonrandom	Hamilton Depression Rating Scale (Ham-D) and Hamilton Anxiety Rating Scale (Ham-A), with cutoff 14	5
Kaur et al^[26]^	Malaysia	Depression, anxiety and stress symptoms among diabetics in Malaysia: a cross-sectional study in an urban primary care setting	1	NR	Primary care government clinics	Face-to-face interview	Two-stage stratified sampling technique	Short version of the Depression, Anxiety and Stress Scale (DASS), i.e., DASS-21 (additional file 1: DASS-21).	9
Mikaliūkštienė et al^[29]^	Lithuania	Prevalence and determinants of anxiety and depression symptoms in patients with type 2 diabetes in Lithuania	1	2007–2010	NC	NC	NC	Hospital Anxiety and Depression Scale (HADS) ≥ 8	4
Alonso-Morán et al^[27]^	Basque Country	Prevalence of depression in adults with type 2 diabetes in the Basque Country: relationship with glycaemic control and health care costs	1	September 1, 2010–August 31, 2011	Basque Country’s public health insurance records	Data extraction from the records	Convenience	Not defined	6
Cho et al^[28]^	Korea	Sleep disturbances and glucoregulation in patients with type 2 diabetes	1	January 2011–April 2011	Diabetic clinics in university hospital	Self-administered	Consecutive	Korean version of the Beck Depression Inventory (K-BDI) Sleep Disorders Questionnaire Sleep Apnea subscale (SDQ-SA) Pittsburgh Sleep Quality Index (PSQI) Korean version of the Epworth Sleepiness Scale (K-ESS)	6
Camara et al^[30]^	Guinea	Prevalence of anxiety and depression among diabetic African patients in Guinea: association with HbA1c levels	1	Between August 2009 and October 2010	Diabetic centers	Semistructured questionnaire and standardized interview	Nonrandom	Hospital Anxiety and Depression Scale (HADS) with cutoff ≥8	6
Foran et al^[31]^	Ireland	Prevalence of depression in patients with type 2 diabetes mellitus in Irish primary care and the impact of depression on the control of diabetes	1	NR	Primary healthcare centers	Secondary data	Convenience	Hospital Anxiety and Depression Scale	5
Wang et al^[34]^	China	Prevalence of depressive symptoms and factors associated with it in type 2 diabetic patients: a cross-sectional study in China	1	NR	Community health center	Interviewer based	Convenience	Zung Self-Rating Depression Scale: depressive symptoms were defined as a standardized score of 53 or higher; 53–62 indicates mild depressive symptoms, 63–72 indicates moderate depressive symptoms, and >72 indicates severe depressive symptoms	7
Lou et al^[33]^	China	Association of sleep quality and quality of life in type 2 diabetes mellitus: a cross-sectional study in China	1	August–December 2012	Healthcare centers	Interviewer based	Multistage random sampling	Pittsburgh Sleep Quality Index (PSQI) (<7, good sleep; ≥7, poor sleep), Self-Rating Anxiety Scale (SAS) and Self-rating Depression Scale (SDS) (≥50 for anxiety and depression)	9
Islam et al^[32]^	Bangladesh	Prevalence of depression and its associated factors in patients with type 2 diabetes: a cross-sectional study in Dhaka, Bangladesh	1	September 2013 and July 2014	Outpatient department of a tertiary care hospital	Face-to-face interview	Consecutive	PHQ-9 cutoff: no or minimal (0–4), mild (5–9), and moderate to severe (≥10) symptoms of depression and severe depression, respectively. Those scores of 0–4 were noted as none to minimal depression.	6
Zhang et al^[35]^	China	Depression in Chinese patients with type 2 diabetes: associations with hyperglycemia, hypoglycemia, and poor treatment adherence	1	July 2010 and July 2011	Outpatient clinics of 6 hospitals	Self-administered	Convenience	PHQ-9 cutoff: >10	8
Chew et al^[36]^	Malaysia	Diabetes-related distress, depression and distress-depression among adults with type 2 diabetes mellitus in Malaysia	1	2012–2013	Public health clinics	Self-administered	Consecutive	PHQ-9	7
Jacob and Kostev^[38]^	Germany	Prevalence of depression in type 2 diabetes patients in German primary care practices	1	January 2004–December 2013	National database	Data extraction from the records	Universal sample	Not defined	7
Sun et al^[40]^	China	Prevalence and determinants of depressive and anxiety symptoms in adults with type 2 diabetes in China: a cross-sectional study	1	August–December 2012	Healthcare centers	NC	Multistage cluster	Pittsburgh Sleep Quality Index (<7, good sleep; ≥7, poor sleep) Zung Self-Rating Anxiety and Depression Scales (≥50)	9
Khullar et al^[39]^	India	The prevalence and predictors of depression in type 2 diabetic population of Punjab	1	October 2011–January 2015	Outpatient clinics of 3 hospitals	Self-administered	Convenience	PHQ-9 (≥10)	5
Handley et al^[37]^	Australia	Suicidal ideation reported by adults with type 1 or type 2 diabetes: results from Diabetes MILES-Australia	1	July 2011	National database of diabetic patients	Self-administered	Simple random	PHQ-9 (≥1 for suicidal ideation, ≥10 for depression)	8
Lee et al^[42]^	Taiwan	Depression and its associated factors among rural diabetic residents	1	2009–2012	Rural areas	Interviewer based	Purposive	Geriatric Depression Scale-Short Form (CGDS-SF)	6
Craike et al^[41]^	Australia	Associations between physical activity and depressive symptoms by weight status among adults with type 2 diabetes: results from Diabetes MILES-Australia	1	July 2011	National database of diabetic patients	Self-administered	Simple random	PHQ-9 (≥10 for depression)	8
Yoshida et al^[45]^	Japan	Association between insomnia and coping style in Japanese patients with type 2 diabetes mellitus	1	NR	Department of Endocrinology and Metabolism at Hirosaki University Hospital	Self-administered	Convenience	Japanese version of the Pittsburgh Sleep Quality Index (PSQI-J): (>5.5) Epidemiological Studies Depression (CES-D): (≥16)	5
Bashkin^[43]^	Europe	Depression among older adults with diabetes in Israel: pattern of symptoms and risk factors	1	2013–2014	Population based	Interviewer based	Multistage cluster	European Depression Scale (>3)	7
Lalwani et al^[50]^	India	Prevalence of depression in Indian patients with type 2 diabetes mellitus and/or hypertension: DEPTH study	1	May–October 2017	Outpatient departments and private clinics	Interviewer based	Convenience	PHQ-9 General Anxiety Disorder questionnaire (GAD-7)	5
Gómez-Peralta et al^[44]^	Mexico	Risk factors and prevalence of suicide attempt in patients with type 2 diabetes in the Mexican population	1	January–December 2016	Diabetes clinic of a hospital	Interviewer based	Convenience	Hamilton Depression Rating Scale:14	5
Alzahrani et al^[47]^	Saudi Arabia	Prevalence and predictors of depression, anxiety, and stress symptoms among patients with type II diabetes attending primary healthcare centers in the western region of Saudi Arabia: a cross-sectional study	1	May and August 2018	Primary healthcare centers	Self-administered	Quota	Depression, Anxiety, and Stress Scale (DASS-21) Depression: ≥10 Anxiety: ≥8 Stress: ≥15	6
Azniza et al^[48]^	Malaysia	Depression and potential risk factors among the elderly with type 2 diabetes mellitus in Kedah, Malaysia	1	Not reported	Single healthcare center	NC	Systematic random	Malay version of Geriatric Depression Scale (M-GDS-14): cutoff ≥5	5
Ding et al^[49]^	Hong Kong	Gender differences in the associations between insomnia and glycemic control in patients with type 2 diabetes: a cross-sectional study	1	July 2010–June 2015	Hong Kong Diabetes Registry	Interviewer based	Consecutive	Insomnia Severity Index (ISI) > 14 EuroQol-5D (EQ-5D): response of “moderate” or “extreme” to the question “anxiety/depression” were considered as having anxiety/depression	7
Alajmani et al^[46]^	Dubai UAE	Prevalence of undiagnosed depression in patients with type 2 diabetes	1	December 2017–November 2018	Diabetic clinics and primary healthcare centers	Face-to-face interviews	Convenience	Beck Depression Inventory (BDI): >16	5
Otaka et al^[51]^	Japan	Association between insomnia and personality traits among Japanese patients with type 2 diabetes mellitus	1	NR	Department of Endocrinology and Metabolism at the Hirosaki University Hospital	Self-administered	Convenience	Center for Epidemiologic Studies Depression Scale (CES‐D): ≥16 Pittsburgh Sleep Quality Index (PSQI-J): current use of hypnotics and ≥5.5	5
DaSantos et al^[53]^	Barbadian	Depression in Barbadian adults with type 2 diabetes	2	NR	Polyclinics	Self-administered	Systematic random	PHQ-9 cutoff ≥10, scores of 5, 10, 15, and 20 represent cut points for mild, moderate, moderately severe, and severe depression, respectively. Those scores of 0–4 were noted as none to minimal depression.	6
Dong et al^[56]^	China	Interaction of sleep quality and anxiety on quality of life in individuals with type 2 diabetes mellitus	1	September 2017	Healthcare centers	Interviewer based	Multistage random	Pittsburgh Sleep Quality Index (PSQI) (<7, good sleep; ≥7, poor sleep), PHQ-9: ≥10 General Anxiety Disorder questionnaire (GAD-7): ≥10	9
Al-Ozairi et al^[52]^	Kuwait	The epidemiology of depression and diabetes distress in type 2 diabetes in Kuwait	1	NR	Diabetes outpatient department and clinical research department	Interviewer based	Convenience	PHQ-9: ≥10	6
Demirci et al^[55]^	Turkey	The screening of comorbid depressive disorders and associated risk factors in adult patients with type 2 diabetes	1	January 2017–May 2018	Tertiary endocrine unit	Self-administered	Consecutive	PHQ-9: ≥10	6
Li et al^[21]^	China	Relationship between illness perception and depressive symptoms among type 2 diabetes mellitus patients in China: a mediating role of coping style	1	June 2018 and September 2019	Department of endocrinology of a hospital	NC	Cluster sampling	Self-rated Depression Scale (SDS): ≥50	7
Whitworth et al^[57]^	Australia	Risk factors and outcomes of anxiety symptom trajectories in type 2 diabetes: the Fremantle Diabetes Study Phase II	1	2008–2016	Community	Face-to-face interviews	Institutional records, advertisement	Generalized Anxiety Disorder Scale (GADS): >15 Generalized Anxiety Disorder—Lifetime Scale (GAD-LT): yes to either of the 2 questions PHQ-9: continuous Brief Lifetime Depression Scale (BLDS): more than 5 symptoms	8
Dehesh et al^[54]^	Iran	Prevalence and associated factors of anxiety and depression among patients with type 2 diabetes in Kerman, Southern Iran	1	August and November 2018	Clinical laboratories	Self-administered and interviewer based	Convenience	Beck Depression Inventory (BDI): ≥18 Hamilton Anxiety (HA) questionnaire: ≥14	5
Sacre et al^[61]^	Australia	Impact of the COVID-19 pandemic and lockdown restrictions on psychosocial and behavioural outcomes among Australian adults with type 2 diabetes: findings from the PREDICT cohort study	1 and 2	May–June 2020	Community (part of an ongoing cohort study)	Telephone interviews	Convenience	Generalized Anxiety Disorder (GAD-7): ≥ 10 Patient Health Questionnaire (PHQ-8): ≥10	7
Niroomand et al^[60]^	Iran	Distress and depression among patients with diabetes mellitus: prevalence and associated factors: a cross-sectional study	1	January and June 2017	Diabetic clinics of 3 hospitals	Self-administered	Convenience	Beck Depression Inventory Second Edition (BDI-II): minimal (0–13), mild (14–19), moderate (20–28), (≥29) symptoms of depression	6
Aschner et al^[58]^	21 developing countries	High prevalence of depressive symptoms in patients with type 1 and type 2 diabetes in developing countries: results from the International Diabetes Management Practices Study	1	2011	Clinics	Self-administered and interviewer based	Convenience	PHQ-9: ≥5	6
Yang et al^[62]^	China	The association between diabetes complications, diabetes distress, and depressive symptoms in patients with type 2 diabetes mellitus	1	July 2016–February 2018	Endocrinology department of a general hospital	Self-administered	Convenience	PHQ-9: ≥10	6
Majumdar et al^[59]^	India	Assessing prevalence and predictors of depression in type 2 diabetes mellitus (DM) patients - The DEPDIAB study	1	NR	Diabetes and endocrinology outpatient department of multiple hospitals in one city	Self-administered	Convenience	PHQ-9 and Becks depression inventory: criteria not stated	6
Chao et al^[20]^	USA	Changes in the prevalence of symptoms of depression, loneliness, and insomnia in U.S. older adults with type 2 diabetes during the COVID-19 pandemic: the Look AHEAD Study	1 and 2	April 2016–June 2018, February 2018–February 2020, July–December 2020	Participants enrolled in Look AHEAD from 2001 to 2004 at 16 sites across the United States	Self-administered	Convenience	PHQ-8 with cutoff ≥10, Women Health Initiative Insomnia rating Scale (Visits 1, 2 and3), only in visit 3 (GAD-7 with cutoff ≥10	6
Ding et al^[63]^	China	The association of the prevalence of depression in type 2 diabetes mellitus with visual-related quality of life and social support	1	July 2012 and May 2013	Community based	Self-administered	Multistage random	CES-D: ≥16	8

NC = not clear, NR = not reported, PHQ = Patient Health Questionnaire.

* Time: 1 = Pre COVID-19, 2 = COVID-19 pandemic.

### 3.3. Study characteristics

Of the studies included, 1 study included data after the COVID-19 pandemic started, and 2 studies reported data from pre- and post-COVID-19 periods. Most of the studies were cross-sectional in design 39 (93%). Matched case–control,^[31]^ cohort, and longitudinal studies were one each.^[38,57]^ Out of 42 studies included, 19 studies were conducted in outpatient departments and endocrinology/diabetes clinics of hospitals. Twelve studies were conducted in primary care centers or other health centers while 1 study did not clarify the setting. Community-based studies were 5. Five studies were based on registries or other records. Most of the studies (29) used nonrandom sampling. 7 studies used multistage cluster or stratified sampling while 4 studies used simple or systematic random sampling. One study did not mention sampling strategy,^[29]^ while one used a universal sample,^[38]^ as shown in Table 1.

A range of tools were used for the assessment of mental health outcomes, that is, anxiety, depression, and sleep disorders. The Patient Health Questionnaire was the most used tool for depression assessment and was used in 15 studies. Other tools used were Beck’s Depression Inventory, General Anxiety and Depression Scale, Hospital Anxiety and Depression Scale, and Depression, Anxiety, and Stress Scale. For sleep disorders, Pittsburg sleep quality indexed was a frequently used tool. Half (21) of the studies collected data by a self-administered questionnaire, while 17 studies employed interviews for data collection, as detailed in Table 1.

### 3.4. Review findings

Sample sizes across studies ranged from 185 to 114,502. However, the sample of 114,502 was in a study based on records.^[27]^ The largest sample in the individual-based study was 7585, which recruited participants from 21 developing countries.^[58]^ The proportion of females in the studies varied, ranging from 31% to 71.4%, as shown in Table 2.

**Table 2:** Summary of finding from included studies.

Study	Sample size	Mean age	Gender, Female, Male	Outcomes	Diabetes duration, range (mean)	Depression, n	Depression, %	Anxiety, n	Anxiety, %	Insomnia, n	Insomnia, %
Khuwaja et al^[24]^	889	NR	57.5, 42.5	Depression, anxiety	NA	387	43.5	515	57.9	xxx	xxx
Tovilla-Zárate et al^[25]^	704	47.39 ± 12.79	55.8, 44.2	Depression, anxiety	NA	340	48.27	388	55.1	xxx	xxx
Kaur et al^[26]^	2508	57 (56.6 ± 10.67	61.1, 38.9	Depression, anxiety	7.7 (6.26)	288	11.5	765	30.5	xxx	xxx
Mikaliūkštienė et al^[29]^	1022	59.3	63.6, 36.4	Depression, anxiety	8.8	291	28.5	433	42.4	xxx	xxx
Alonso-Morán et al^[27]^	114,502	NR	43.3, 56.7	Depression	NR	12,392	9.8	xxx	xxx	xxx	xxx
Cho et al^[28]^	614	59.7 ± 11.1	37.9, 62.1	Depression Insomnia Sleep apnea Daytime sleepiness	10.3 ± 8.4	175	28.5	xxx	xxx	296	48.2
Camara et al^[30]^	491	57.9	62.7, 37.3	Depression, anxiety	NR	169	34.4	288	58.7	xxx	xxx
Foran et al^[31]^	283 + 283	68 (9.5)	42, 58	HADS score	NR	63	22	xxx	xxx	xxx	xxx
Wang et al^[34]^	865	70.13 (±20.33)	53.4, 46.6	Depression	NR	304	35.1	xxx	xxx	xxx	xxx
Lou et al^[33]^	944	64.1 ± 10.2	61.3, 38.9	Depression, anxiety, Sleep quality	5.6 ± 5.1	379	40.1	618	65.5	6.87 ± 3.84	xxx
Islam et al^[32]^	515	49.94 ± 10.21	55.92, 44.08	Depression	Median (interquartile range) for diabetes duration was 3.0 (6.00) years	319	61.9	xxx	xxx	xxx	xxx
Zhang et al^[35]^	2538	56.4 ± 10.5	47, 53	Depression	Median (interquartile range): 6 (2–10)	155	6.1	xxx	xxx	xxx	xxx
Chew et al^[36]^	700	56.9	52.8, 47.2	Depression	Median (interquartile range) for diabetes duration was 4.0 (6.00) years	292	41.7	xxx	xxx	xxx	xxx
Jacob and Kostev^[38]^	90,412	65.5 ± 11.7	49.8, 50.2	Depression	NR	xxx	1 year: 5.9 5 years: 17.7 10 years: 30.0	xxx	xxx	xxx	xxx
Sun et al^[40]^	893	63.9 ± 10.2	58.6, 41.2	Depression, anxiety, Sleep quality	5.6 ± 5.1	389	43.6	501	56.1	305	34.2
Khullar et al^[39]^	787	Men: 54.9 ± 9.8 Women: 53.8 ± 11.2	49.05. 50.95	Depression	NR	Mild to moderate: 199 Severe: 252	Mild to moderate: 25.32 Severe: 32.02	xxx	xxx	xxx	xxx
Handley et al^[37]^	1962	58.55 ± 8.71	49.1, 50.9	Depression, suicidal ideation	NR	522	26.6	xxx	xxx	xxx	xxx
Lee et al^[42]^	696	Depressive: 69.7 (8.9) Nondepressive: 67.9 (9.6)	58.3, 41.7	Depression	Depressive: 8.3 (6.5) Nondepressive: 9.0 (6.6)	117	16.8	xxx	xxx	xxx	xxx
Craike et al^[41]^	705	59 ± 8	50, 50	Depression	8.6 (6.7)	195	28	xxx	xxx	xxx	xxx
Yoshida et al^[45]^	503	63.9 ± 12.5	42, 58	Depression, Insomnia	NR	xxx	xxx	xxx	xxx	141	28
Bashkin^[43]^	561	Depressive: 71.9 ± 9.75 Nondepressive: 68.2 ± 8.69	Not clear	Depression	NR	NR	42.6% women 24.8% men	xxx	xxx	xxx	xxx
Lalwani et al^[50]^	631	49.95 ± 8.25	50.1, 49.9	Depression, anxiety	NR	322	51.03	322	51.03	xxx	xxx
Gómez-Peralta et al^[44]^	185	54.1 ± 13.2	71.4, 28.6	Depression, suicidal ideation	NR	32	17.3	xxx	xxx	xxx	xxx
Alzahrani et al^[47]^	450	56.9 ± 11.1	43.1, 56.9	Depression, anxiety, stress	Median (range): 8 (75)	153	33.8	171	38	xxx	xxx
Azniza et al^[48]^	511	64.5 ± 7.0	46.2, 53.8	Depression	8.57 ± 5.57	164	32.1	xxx	xxx	xxx	xxx
Ding et al^[49]^	3753	Noninsomniac: 54.3 (8.6) Insomniac: 54.2 (8.3)	42.6, 57.4	Depression, anxiety, insomnia	Noninsomniac: 7.0 (3.0–13.0) Insomniac: 8.0 (3.0–15.0)	Anxiety/depression: 639	17	xxx	xxx	359	9.6
Alajmani et al^[46]^	559	NR	43, 57	Depression	NR	95	17	xxx	xxx	xxx	xxx
Otaka et al^[51]^	504	63.9 ± 12.5	41.8, 58.2	Depression, insomnia	NR	Reported mean scores of CES-D from insomnia and noninsomnia participants	xxx	xxx	xxx	154	30.6
DaSantos et al ^[53]^,*	509	63.54	352, 157	Depression	13.51 (SD = 10.55)	NR	9.4	0	0	0	xxx
Dong et al^[56]^	944	64.1 ± 10.2	61.3, 38.9	Depression, anxiety, sleep quality	5.6 ± 5.1	NR	NR	652	65.4	335	33.6
Al-Ozairi et al^[52]^	465	55.3 ± 10.1	48.2, 52.8	Depression	12.5 ± 8.2	136	29.2	xxx	xxx	xxx	xxx
Demirci et al^[55]^	460	59.48 ± 9.72	58, 42	Depression	NR	87	18.9	xxx	xxx	xxx	xxx
Li et al^[21]^	939	41.08 ± 12.27	40.8, 59.2	Depression	NR	691	73.59	xxx	xxx	xxx	xxx
Whitworth et al^[57]^	1091	64.8 ± 10.8	46.1, 53.9	Anxiety, depression	NR	393	36.00	137	12.6	xxx	xxx
Dehesh et al^[54]^	1500	47.12 ± 12.52	51.3, 48.7	Anxiety, depression	NR	885	59	930	62	xxx	xxx
Sacre et al ^[61]^,†	450	66 ± 9	31, 69	Anxiety, depression	NR	Pre: 24 Post: 25	Pre: 5.3 Post: 5.6	Pre: 38 Post: 38	8.4 8.4	xxx	xxx
Niroomand et al^[60]^	820	58.91 ± 12.35	64.2, 35.8	Depression	11.53 ± 6.89	Minimal: 147 Mild: 117 Moderate: 181 Severe: 81	20.2 16.1 24.9 38.7	xxx	xxx	xxx	xxx
Aschner et al^[58]^	7585	56.8 (11.3) to 60.08 (11.52)	52.7, 47.3	Depression	6.8 ± 6.1 to 12.6 ± 8.6	2514	33.1	xxx	xxx	xxx	xxx
Yang et al^[62]^	600	57.17 ± 9.75	62.17, 37.83	Depression	9.75 ± 7.00	166	27.67	xxx	xxx	xxx	xxx
Majumdar et al^[59]^	1371	NR	39.02, 60.9	Depression	NR	550	40.26	xxx	xxx	xxx	xxx
Chao et al ^[20]^,†	2829	Pre = 74.1 (6.0) Post = 75.6 (6.0)	63.2, 36.8	Insomnia and depression pre/post COVID-19, anxiety after COVID-19	NR	Pre: 454 Post: 715	Pre:19.3 Post:30.4	565	20	Pre: 848 Post: 801	Pre:33.3 Post:31.4
Ding et al^[63]^	1618	61.69 ± 8.72	61, 39	Depression	7.65 ± 5.93	378	23.36	xxx	xxx	xxx	xxx

CES-D = Epidemiological Studies Depression, HADS = Hospital Anxiety and Depression Scale, NR = not reported, SD = standard deviation, xxx = outcome not assessed or not reported in the original study.

* Pandemic era study.

† Pre- and postpandemic study on same population.

**Figure 2.**
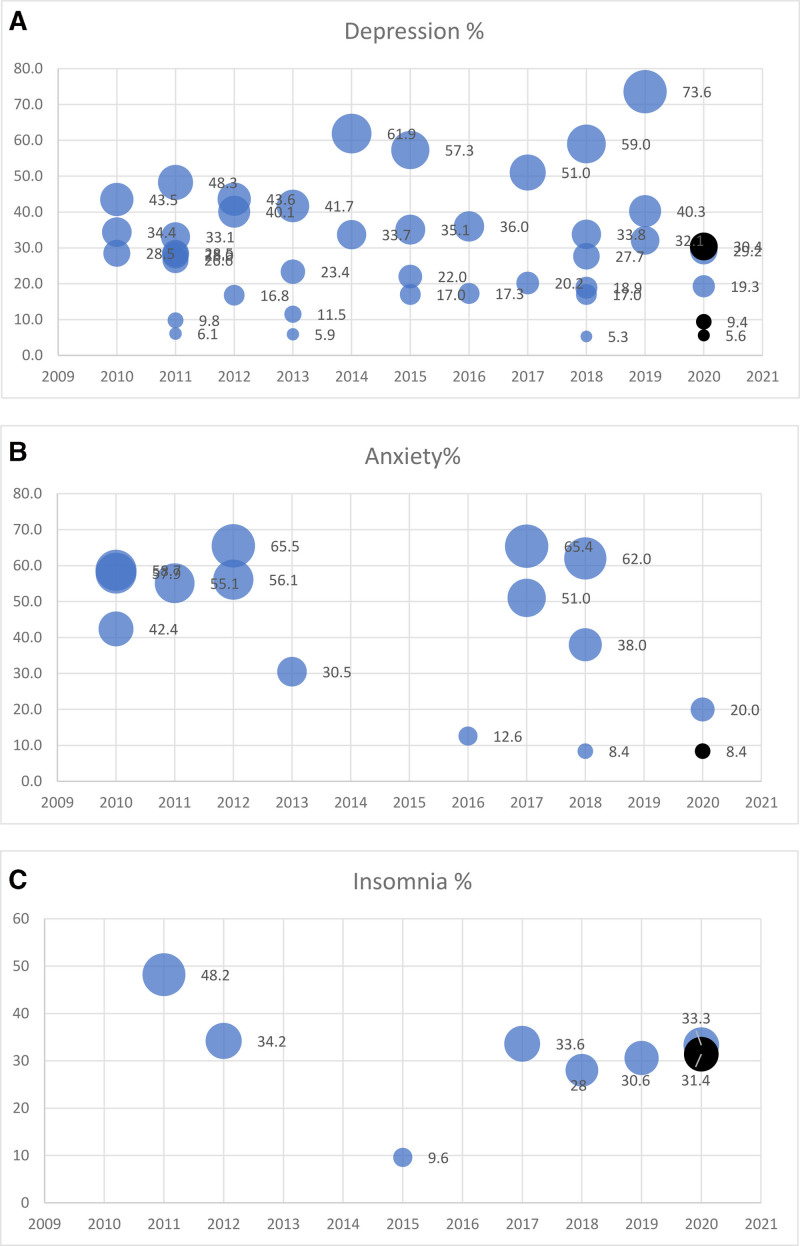
Prevalence of depression (A), anxiety (B), and insomnia (C) reported in included studies (blue = prepandemic, black = during pandemic).

Thirty-nine studies reported depression as one of the outcomes. The prevalence of depression reported in all the included studies and the studies conducted before the pandemic ranged from 5.3% to 73.6%. Among the studies that reported depression during the COVID-19 pandemic, the prevalence ranged from 5.6% to 30.4%.^[20,53,61]^ Of the 2 studies that compared depression prevalence in prepandemic and pandemic periods in the same population, one study did not find a significant difference (5.3% vs 5.6%).^[61]^ Another study reported a significant increase from 19.3% between February 2018 and 2020 to 30.4% between July and December 2020^[20]^ (Fig. 2A).

Anxiety was reported in 14 studies. The overall prevalence of anxiety ranged from 8.4% to 65.5% in 13 studies conducted before the pandemic. Only one study compared prepandemic and pandemic periods’ prevalence of anxiety and did not find any difference in the prevalence; 8.4% and 8.4%^[61]^ (Fig. 2B). Insomnia was reported in 7 studies out of which 1 reported a mean score instead of a binary outcome. Prevalence in 5 studies, conducted before the pandemic, ranged from 9.6% to 48.2%. A study comparing prevalence before the pandemic and pandemic times found a slight reduction from 33.3% to 31.4%^[20]^ (Fig. 2C). The suicidal intention was reported in 2 studies conducted before the pandemic and the prevalence was 15.9%^[37]^ and 11.3%.^[44]^

Risk factors of depression and anxiety reported in the included studies are summarized in Table 3. Most reported risk factors for depression were female gender, age, low socioeconomic status, presence of comorbidities, duration of diabetes, poor glycemic control, deranged lipid profile, diabetes complications, obesity, and physical inactivity. Risk factors for anxiety were more or less similar to those of depression and included female gender, age, low socioeconomic status, financial hardship, poor glycemic control, diabetes complications, and family problems.

**Table 3:** Risk factors of depression and anxiety.

Study	Depression	Anxiety
Khuwaja et al^[24]^	Female gender, older age, hypertension, ischemic heart disease, increase BMI, fasting BG, fasting blood triglycerides	Physical inactivity, hypertension, ischemic heart disease, systolic blood pressure, fasting BG, fasting blood triglycerides
Tovilla-Zárate et al^[25]^	Glucose level and diabetes complications	Occupation and diabetes complications
Kaur et al^[26]^	Females, Asian Indians, marital status, family history of psychiatric illness, less than 2 years duration of diabetes and current alcohol consumption	Unemployment, being housewives, HbA1c level of more than 8.5%, family history of psychiatric illness, life events and lack of physical activity
Mikaliūkštienė et al^[29]^	Old age and duration of diabetes	Old age, low education, while collar work, duration of diabetes, and complications
Alonso-Morán et al^[27]^	NR	NR
Cho et al^[28]^	Female gender and age > 65 years	NR
Camara et al^[30]^	Women: Urban residence, older age, low SES, no previous measure of HbA1c Men: Insulin therapy and HbA1c ≥ 9	Women: Urban residence Men: Low SES, and high HbA1c
Foran et al^[31]^	Poor glycemic control	NR
Wang et al^[34]^	NR	Sleeping < 7 hours, history of MI, being employed, high total cholesterol
Lou et al^[33]^	NR	NR
Islam et al^[32]^	NR	Age less than 60, female gender, having complications, loss in business, family conflicts, separation from family, unavailability of food or medicine
Zhang et al^[35]^	NR	NR
Chew et al^[36]^	Microvascular complications; retinopathy, nephropathy and diabetic foot problems, higher DDS (diabetes-related distress)	NR
Jacob and Kostev^[38]^	Female gender, no private health insurance, neuropathy, retinopathy, CHD, stroke, HbA1c > 7	NR
Sun et al^[40]^	NR	NR
Khullar et al^[39]^	Female, high glucose, age more than 30 years, low income, sedentary life style, alcohol consumption, use of statins, high BMI	NR
Handley et al^[37]^	NR	NR
Lee et al^[42]^	Lack of sleep, lack of exercise, and sedentary life style	NR
Craike et al^[41]^	Low physical activity	NR
Yoshida et al^[45]^	NR	NR
Bashkin^[43]^	Female gender, financial difficulty, activity limitations	NR
Lalwani et al^[50]^	Overweight and obesity, smoking, upper middle income	NR
Gómez-Peralta et al^[44]^	NR	NR
Alzahrani et al^[47]^	Female gender, younger age, duration of diabetes, comorbidities, HbA1c, noncompliance with medication	Female, comorbidities, family history of chronic diseases, most recent HbA1c level, noncompliance
Azniza et al^[48]^	Increasing HBA1c, DM complications	NR
Ding et al^[49]^	NR	NR
Alajmani et al^[46]^	Attending mini diabetic clinic vs general family practice clinic, age, female gender, lower education and unemployment	NR
Otaka et al^[51]^	NR	NR
DaSantos et al^[53]^,*	Younger age less than 45, increasing HbA1c level, female gender	NR
Dong et al^[56]^	NR	NR
Al-Ozairi et al^[52]^	Gender, past history of depression, psychological distress	NR
Demirci et al^[55]^	Female, smoking	NR
Li et al^[21]^	Presence of complications	NR
Whitworth et al^[57]^	NR	NR
Dehesh et al^[54]^	Female, obesity, high FBG, high LDL, low HDL, high triglycerides, high HBA1c, hypertension, and lack of physical activity	NR
Sacre et al ^[61]^,†	NR	NR
Niroomand et al^[60]^	NR	NR
Aschner et al^[58]^	Female, complications, low SES	NR
Yang et al^[62]^	Physical activity, diabetic complications	NR
Majumdar et al^[59]^	Female, low SES, high FBS and HbA1c, hospitalization, low BMI	NR
Chao et al ^[20]^,†	Female, non-Hispanic White, obesity, history of depression during previous visits	Younger age, female
Ding et al^[63]^	Age < 60, lack of social support, poor visual QoL	NR

BG = blood glucose, BMI = body mass index, CHD = coronary heart disease, DM = diabetes mellitus, FBG = fasting blood glucose, HbA1c = hemoglobin A1c, HDL = high-density lipoprotein, LDL = low-density lipoprotein, NR = not reported, SES = socioeconomic status, QoL = quality of life

* Pandemic era study.

† Pre- and postpandemic study on same population.

## 4. Discussion

COVID-19 has affected all segments of life in an unprecedented way. People with chronic diseases were at high risk of the direct and indirect effects of the pandemic. In this systematic review, 42 studies were included to assess the impact of COVID-19 on the mental health of type 2 diabetic patients before and during the COVID-19 era.

In this review, we found that the prevalence of depression ranged from 5.3% to 73.6%. A wide variation in the prevalence of depression among individuals with type 2 diabetes has also been reported in a systematic review and meta-analysis of depression worldwide. It was found that the prevalence ranged from 2% to 88%.^[65]^ Similar findings were reported in a meta-analysis of Chinese studies, where the prevalence ranged from 0.4% to 52%.^[66]^ Another meta-analysis of studies involving Indian type 2 diabetic patients reported a prevalence of depression ranging from 10% to 88%.^[67]^

The wide variation in the prevalence of depression among individuals with type 2 diabetes can be attributed to differences in the assessment tools used for diabetes, cutoff points, study settings, designs, and population characteristics. A meta-analysis found varying prevalence rates of anxiety and depression depending on the assessment tools used.^[66]^

Nevertheless, in a study that compared depression rates in the same participants before and during the pandemic, Chao et al^[20]^ identified a significant change, with rates increasing from 19.3% before the pandemic to 30.4% during it. Moradian et al^[68]^ supported these findings, emphasizing the considerable mental health impact of COVID-19 on this population. Several factors can explain this shift in depression rates. For instance, Rosenbaum^[69]^ highlighted widespread disruptions in routine medical care as healthcare resources were increasingly redirected toward addressing COVID-19. Additionally, global supply chain disturbances resulted in medication shortages, presenting additional challenges for those dependent on regular medication.^[70]^ Furthermore, Holmes et al^[71]^ underscored the heightened isolation experienced by those considered high-risk as they took precautions to avoid contracting the virus. This isolation, combined with the anxiety of potentially severe health outcomes if infected, further strained the mental health of individuals with preexisting conditions.^[72]^

In the studies reviewed, anxiety prevalence in type 2 diabetes patients before the COVID-19 pandemic ranged from 8.4% to 65.5%. During the pandemic, this range was 8.4% to 20%. Sacre et al compared anxiety levels from both periods and found no significant difference. This observation contrasts with other research suggesting increased anxiety levels during the pandemic. Many of these studies collected data during the pandemic’s early stages and often focused on general mental health, not specifically on type 2 diabetes.^[61]^

Robinson et al conducted a meta-analysis on mental health during the pandemic. Their findings indicated a temporary increase in anxiety and depression after the pandemic’s onset, but levels soon returned to prepandemic norms. Given the limited research comparing anxiety levels in type 2 diabetes patients before and during the pandemic, further investigations are warranted.^[73]^

In our systematic review, Chao et al^[20]^ stands out as the only study that compared insomnia prevalence among type 2 diabetes patients during both prepandemic and pandemic contexts, revealing no significant change. However, this result is inconsistent with an international study.^[74]^ They observed an elevated incidence of insomnia during the pandemic’s onset. Specifically, their reported rates were nearly double those of established nonpandemic research.^[75]^ The discrepancy between these studies invites further reflection. Several factors might account for the divergent findings. First, the sample populations and regional variations might play a role; perhaps Chao et al cohort faced different pandemic-induced stressors. Second, methodological differences, such as the criteria used to diagnose insomnia, could lead to variance in reported rates.^[20]^ The timing and phase of the pandemic when the data were collected might also have had differential impacts on the participants’ mental health. Additionally, external societal and healthcare factors during the pandemic could influence insomnia rates.

Although it was hypothesized that the COVID-19 pandemic would significantly worsen mental health outcomes among diabetic patients, this study’s findings suggest a more nuanced situation. The anticipated increase in the prevalence of mental health disorders, such as anxiety and insomnia, was not as pronounced as expected, indicating a complex interplay of factors that warrants further investigation to fully understand the pandemic’s impact on this vulnerable population.^[20,61,76]^ This finding may be partly attributed to reduced access to healthcare services during the pandemic, which could have impacted the reporting and diagnosis of mental health issues. Additionally, the systematic review and meta-analysis by Robinson et al provides further insight into the trajectory of mental health issues during the pandemic. Their review identified an initial surge in the prevalence of mental health problems, followed by a gradual return to prepandemic levels by mid-2020. These observations highlight the complex dynamics at play and underscore the need for ongoing research to better understand the pandemic’s long-term effects on mental health, particularly in vulnerable populations such as individuals with type 2 diabetes.^[73]^

Interestingly, the data only demonstrated a notable change in the case of depression, as one study revealed a significant increase in depression rates when comparing the period before and during the COVID-19 pandemic among individuals with diabetes.^[20]^ This finding suggests that depression might be a mental health concern more susceptible to exacerbation in this population during this challenging period.

Several risk factors for mental health issues such as anxiety and depression have been reported in the literature. These include female gender, young age, low education, low income, occupation, ethnicity, lack of physical activity, comorbidities, lack of social support, previous and family history of mental illness, and poor glycemic control.^[66,77–79]^ In addition to these, COVID-19-related worriedness and fear were also reported in recent studies.^[68,78]^

### 4.1. Limitations

This review synthesized evidence from a wide range of studies using standard guidelines and procedures. However, certain limitations need to be considered while interpreting the results of this review. First, all the studies were observational, therefore, causal inferences between pandemic and mental health issues cannot be drawn with certainty. Second, the number of studies reporting pandemic-era mental health status was very few, which makes comparison with prepandemic time difficult. Furthermore, before and after comparisons on the same population/patients were even fewer. Third, although valid, a wide range of tools are used in epidemiological studies to assess depression and anxiety, which makes comparisons among the studies less reliable. However, this is an implicit issue in systematic reviews and meta-analyses for any health outcome such as depression and anxiety with several tools for assessment. Fourth, mental health is also affected by socioeconomic and other external factors of patients, which were not taken into consideration in the included studies. Fifth, because of the limited resources available and the linguistic competency of the authors, only studies published in English evaluated. However, we assume it to be a minimal limitation as most of the literature published is in English and we included studies from a wide range of countries and regions.

## 5. Conclusions

Our systematic review examined the impact of the COVID-19 pandemic on the mental health of individuals with type 2 diabetes. While our study contributed valuable insights, the limited number of relevant studies directly comparing mental health during COVID-19 with prepandemic baseline data may affect the precision of our findings. Therefore, conducting longitudinal research is essential to gain a better understanding of the intricate relationship between the pandemic, mental health, and type 2 diabetes. This knowledge is critical for developing tailored clinical interventions and evidence-based policies to support individuals with type 2 diabetes during global health crises.

## Author contributions

**Conceptualization:** Amani Busili, Unaib Rabbani.

**Data curation:** Amani Busili.

**Formal analysis:** Amani Busili.

**Methodology:** Amani Busili, Kanta Kumar, Laura Kudrna, Unaib Rabbani.

**Writing – original draft:** Amani Busili, Unaib Rabbani.

**Writing – review & editing:** Amani Busili, Kanta Kumar, Laura Kudrna, Unaib Rabbani.

**Supervision:** Kanta Kumar, Laura Kudrna.

**Validation:** Kanta Kumar, Laura Kudrna, Unaib Rabbani.

**Investigation:** Laura Kudrna.

**Visualization:** Laura Kudrna.
